# Models of ultrasonic radiomics and clinical characters for lymph node metastasis assessment in thyroid cancer: a retrospective study

**DOI:** 10.7717/peerj.14546

**Published:** 2023-01-12

**Authors:** Hui Zhu, Bing Yu, Yanyan Li, Yuhua Zhang, Juebin Jin, Yao Ai, Xiance Jin, Yan Yang

**Affiliations:** 1 Department of Ultrasound, the Second Affiliated Hospital of Wenzhou Medical University, Wenzhou, Zhejiang, China; 2 Department of Radiotherapy Center, The 1st Affiliated Hospital of Wenzhou Medical University, Wenzhou, Zhejiang, China; 3 Department of Medical Engineering, The 1st Affiliated Hospital of Wenzhou Medical University, Wenzhou, Zhejiang, China

**Keywords:** Ultrasound, Radiomics, Machine learning, Lymph node metastasis, Papillary thyroid carcinoma

## Abstract

**Background:**

Preoperative prediction of cervical lymph node metastasis in papillary thyroid carcinoma provided a basis for tumor staging and treatment decision. This study aimed to investigate the utility of machine learning and develop different models to preoperatively predict cervical lymph node metastasis based on ultrasonic radiomic features and clinical characteristics in papillary thyroid carcinoma nodules.

**Methods:**

Data from 400 papillary thyroid carcinoma nodules were included and divided into training and validation group. With the help of machine learning, clinical characteristics and ultrasonic radiomic features were extracted and selected using randomforest and least absolute shrinkage and selection operator regression before classified by five classifiers. Finally, 10 models were built and their area under the receiver operating characteristic curve, accuracy, sensitivity, specificity, positive predictive value and negative predictive value were measured.

**Results:**

Among the 10 models, RF-RF model revealed the highest area under curve (0.812) and accuracy (0.7542) in validation group. The top 10 variables of it included age, seven textural features, one shape feature and one first-order feature, in which eight were high-dimensional features.

**Conclusions:**

RF-RF model showed the best predictive performance for cervical lymph node metastasis. And the importance features selected by it highlighted the unique role of higher-dimensional statistical methods for radiomics analysis.

## Introduction

Thyroid cancer is commonly seen in clinics and it ranked the top 10 major cancer in China with an incidence and mortality rate of 1.00% and 0.34% in 2015 (Du et al., 2020). Moreover, in province like Zhejiang, its incidence rate rises to the top of all cancers in females (Du et al., 2020). Papillary thyroid carcinoma (PTC) is the most common histological type of thyroid malignancy. Although it presents with indolent procedure, recurrence and metastasis are unavoidable. Literature showed a strong correlation between cervical lymph node metastasis (LNM) and recurrence or poor survival rate in PTC (Hartl et al., 2012; Kai Guo, 2014; Zhou et al., 2020a). In addition, the judgement of cervical LNM affects the staging of PTC as well as its treatment and the extent of resection (Haugen et al., 2016; Kim et al., 2017).

Cervical LNM presents in 20%–90% patients at diagnosis with an incidence of 30%–65% and 3%-44.5% for central LNM (level VI) and lateral LNM (level II-V), respectively (Hartl et al., 2012; Tong et al., 2020). High-resolution ultrasound (US) is the first-line noninvasive imaging method in detecting PTC (Jiang et al., 2020). However, the diagnostic value of US in cervical LNM is limited with high specificity (85.0%–97.4%) but low sensitivity (36.7%–61.0%) (Jiang et al., 2020). Radiomics analysis quantitatively extracts high-throughput features from medical images and converts them into mineable data to help diagnosing or predicting diseases in clinical practice (Jiang et al., 2020; Lu et al., 2019). Recent studies showed that radiomic features of magnetic resonance imaging (MRI), computed tomography (CT) images had some potential predicting values in cervical LNM in patients with thyroid carcinoma (Hu et al., 2020; Zhou et al., 2020b). Similar results were found in US images for predicting central or lateral LNM (Jiang et al., 2020; Park et al., 2020b).

Machine learning (ML), which acts like a subset of artificial intelligence, has become the top interest in medical imaging recently (Shin et al., 2020). It is comprised of multiple computational models and methods using meaningful features extracted from medical image, and thus can draw results with consistent diagnostic and prognostic accuracy (Lee et al., 2020; Shin et al., 2020). Recent studies based on ML method had been applied to thyroid US imaging (Shin et al., 2020; Zhao et al., 2021). However, little study has applied ML to analyzing the predicting value for cervical LNM in PTC nodules based on the radiomic features extracted from US images.

The purpose of this study was to investigate the utility of ML and develop 10 models to preoperative predict cervical LNM based on US radiomic features and clinical characteristics in PTC nodules.

## Materials & Methods

The authors are accountable for all aspects of the work in ensuring that questions related to the accuracy or integrity of any part of the work are appropriately investigated and resolved. The study was conducted in accordance with the Declaration of Helsinki (as revised in 2013). The study was approved by institutional ethics committee of the Second Affiliated Hospital of Wenzhou Medical University (NO.: 2021-K-20-01). Informed consent was waived by the local Ethics Committee in view of the retrospective nature of the study and all the procedures being performed were part of the routine care.

### Patients, clinical characteristics and surgery

**Figure 1:**
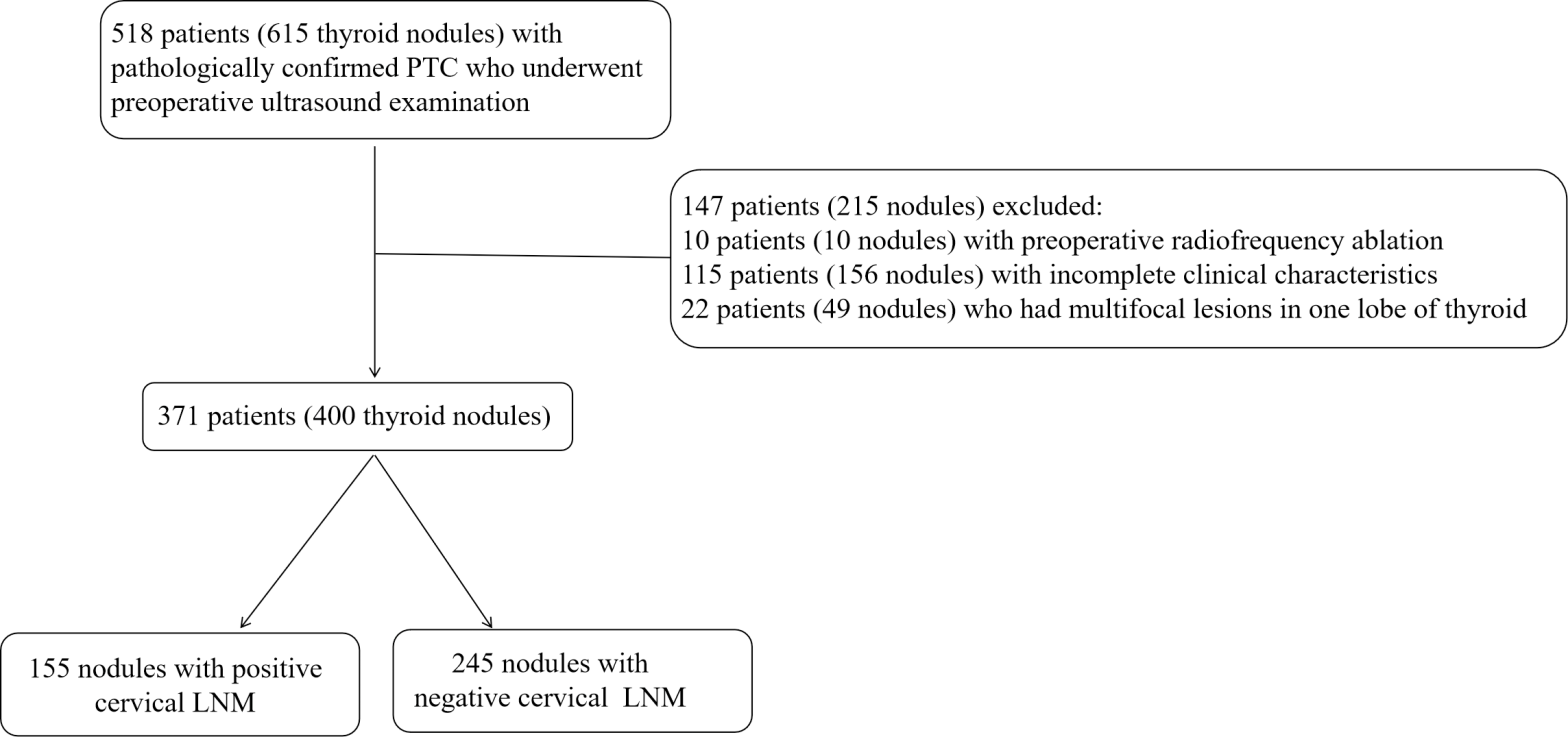
The flowchart of nodules’ inclusion and exclusion for this retrospective study. LNM, lymph node metastasis; PTC, Papillary thyroid carcinoma; US, ultrasound.

From January 2018 to September 2019, 615 nodules from 518 consecutive patients were involved in this study. Among them, four patients had four nodules, 12 patients had three nodules and 61 patients had two nodules on contralateral or ipsilateral lobe of thyroid. Our study was based on nodule level. The inclusion criteria for each nodule included: (1) preoperative US examination; (2) initial surgical resection within 2 weeks of US examination; (3) nodules confirmed with PTC pathologically; (4) cervical dissection and lymph node resection with pathologically confirmation of state. The exclusion criteria for each nodule included: (1) preoperative radiofrequency ablation; (2) incomplete clinical characteristics; (3) multifocal lesions in one lobe of the thyroid. In total, 400 nodules from 371 patients were included in this study. Among them, 58 nodules were from separate lobe of thyroid of 29 patients, *i.e.*, each patient had one nodule on each side of thyroid. The cohort consists of 101 males and 270 females with the mean age of 45.73 ± 11.84 years (ranging from 12 to 81 years). The flowchart of nodules’ inclusion and exclusion was shown in Fig. 1.

**Figure 2:**
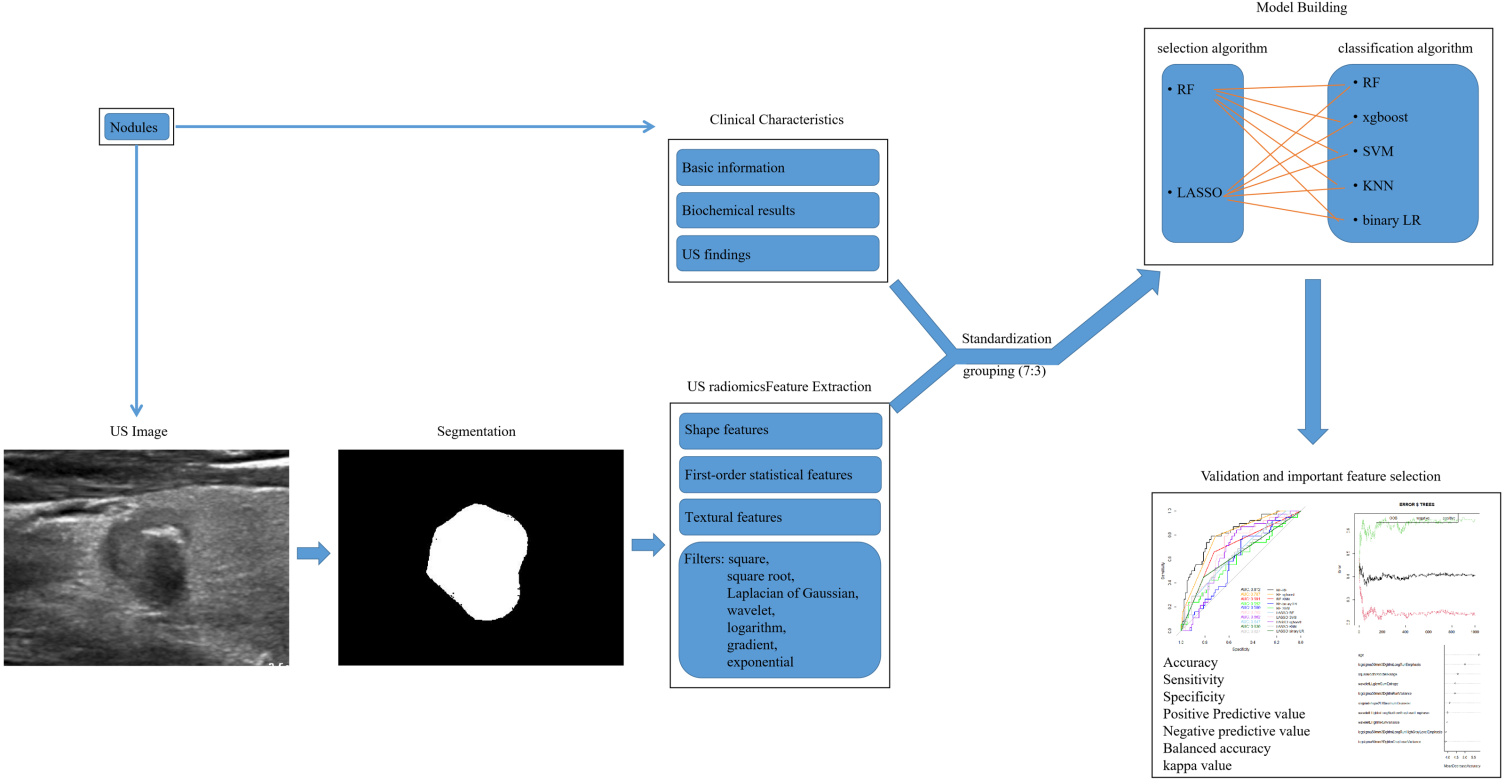
The workflow of US radiomic feature extraction, model building and validation in this retrospective study. US, ultrasound; RF, randomforest; LASSO, the least absolute shrinkage and selection operator; xgboost, the extreme gradient boosting; SVM, support vector machine; KNN, k nearest neighbors; LR, logistics regression.

Clinical characteristics included basic information (age and sex), biochemical results and US findings. Standardized biochemical examination was done including differential blood count, liver function analysis, renal function analysis, blood calcium ion, routine urianlysis, serum free triiodothyronine 3 (FT3), serum FT4, serum total triiodothyronine 3 (TT3), serum TT4, serum thyroid stimulating hormone (TSH), serum anti-thyroglobulin antibodies (ANTITGAB), serum anti-thyroid peroxidase antibody (ANTITPOAB) and serum thyroglobulin (TG). US findings of composition, echogenicity, shape, margin and echogenic foci were measured, scored and classified according to the Thyroid Imaging Reporting and Data System (TI-RADS) criteria of American College of Radiology (Haugen et al., 2016). In total, there were 34 clinical characteristics (Table 1, Fig. 2).

Total or near total thyroidectomy or hemithyroidectomy was performed according to the clinical TNM staging with prophylactic or therapeutic cervical dissection and lymph node dissection. Cervical LNM were determined when ipsilateral lymph nodes were proved metastasis pathologically. It was because tumor cells metastasized to central lymph node, followed by lateral lymph node. Rarely, skip’ lesions could occur (Table 1) (Al Afif et al., 2015).

**Table 1:** Clinical characteristics of nodules in training and validation groups.

Characteristics	Training group (*n* = 282)	Validation group (*n* = 118)	*p* value
Sex			0.240
Male	70(24.82)	36(30.51)	
Female	212(75.18)	82(69.49)	
Age (years) †	45.66 ± 12.03	46.65 ± 11.76	0.449
Size (mm)	9.97 ± 5.95	10.03 ± 7.57	0.410
WBC ($\times 10\hat {}$9/L)	6.11 ± 1.56	5.94 ± 1.57	0.302
NEUT ($\times 10\hat {}$9/L)	3.75 ± 1.27	3.67 ± 1.28	0.478
LYM ($\times 10\hat {}$9/L)	1.91 ± 0.59	1.84 ± 0.50	0.448
HB (g/L)	138.18 ± 15.98	141.63 ± 14.33	0.032
RBC ($\times 10\hat {}$12/L)	4.66 ± 0.45	4.72 ± 0.44	0.336
PLT ($\times 10\hat {}$9/L)	258.34 ± 62.73	242.37 ± 53.28	0.066
ALT (U/L)	23.59 ± 20.03	22.19 ± 16.91	0.743
AST (U/L)	21.32 ± 8.09	20.87 ± 6.75	0.956
ALB (g/L)	45.09 ± 3.08	45.19 ± 3.04	0.742
BUN (mmol/L)	4.85 ± 1.26	4.87 ± 1.33	0.919
CREA (umol/L)	57.59 ± 13.33	57.81 ± 12.04	0.428
UA (umol/L)	313.35 ± 78.17	321.46 ± 92.40	0.754
Ca (mmol/L) †	2.40 ± 0.10	2.40 ± 0.12	0.955
TT3 (ng/ml)	1.08 ± 0.17	1.12 ± 0.40	0.426
TT4 (*μ*g/dl)	8.24 ± 1.55	8.31 ± 1.92	0.998
FT3 (pg/ml)	3.28 ± 0.40	3.47 ± 1.58	0.174
FT4 (ng/dl)	1.29 ± 0.17	1.32 ± 0.34	0.476
TSH (*μ*IU/ml)	1.71 ± 1.05	1.83 ± 1.34	0.902
ANTITGAB (IU/ml)	117.99 ± 327.15	130.06 ± 348.83	0.817
ANTITPOAB (IU/ml)	39.65 ± 94.90	50.47 ± 126.10	0.818
TG (ng/ml)	37.97 ± 80.06	28.13 ± 39.32	0.747
Urinary leukocyte§			0.516
Negative	229(81.21)	100(84.75)	
Positive 1+	22(7.80)	9(7.63)	
Positive 2+	15(5.32)	6(5.08)	
Positive 3+	12(4.26)	1(0.85)	
Positive 4+	4(1.42)	2(1.69)	
URBC§			0.323
Negative	253(89.72)	104(88.14)	
Positive 1+	21(7.45)	8(6.78)	
Positive 2+	5(1.77)	3(2.54)	
Positive 3+	0(0.00)	2(1.69)	
Positive 4+	3(1.06)	1(0.85)	
Urinary protein			0.296
Negative	171(60.64)	80(67.80)	
Positive 1+	77(27.30)	29(24.58)	
Positive 2+	34(12.06)	9(7.63)	
Composition§			1.000
Predominately cystic	2(0.71)	1(0.88)	
Predominately solid	279(98.94)	117(99.12)	
Solid	1(0.35)	0(0.00)	
Echogenicity§			0.971
Hyperechoic or isoechoic	9(3.19)	4(3.39)	
Hypoechoic	231(81.91)	98(83.05)	
Markedly hypoechoic	42(14.89)	16(13.56)	
Shape			0.253
Wider-than-tall	125(44.33)	45(38.14)	
Taller-than-wide	157(55.67)	73(61.86)	
Margin			0.615
Smooth or ill-defined	153(54.26)	69(58.47)	
Lobulated or irregular	93(32.98)	33(27.97)	
Extrathyroidal extension	36(12.77)	16(13.56)	
Echogenic foci§			0.025
No calcification	67(19.76)	32(9.44)	
Macrocalcifications	84(24.78)	17(5.01)	
Peripheral calcifications	6(1.77)	1(0.29)	
Microcalcifications	182(53.69)	81(23.89)	
TI-RADS score	9.17 ± 2.41	9.16 ± 2.28	0.943
TI-RADS classification§			0.499
III	1(0.35)	0(0.00)	
IV	19(6.74)	10(8.47)	
V	262(92.91)	108(91.53)	
Cervical LNM			0.082
Negative	165(58.51)	80(67.80)	
Positvie	117(41.49)	38(32.20)	

**Notes.**

Continuous variables were presented with Mean ±SD while categorical variable were presented with number and percentage (percentage in parentheses). Continuous variables were calculated by Mann–Whitney U test except for data marked with †, which were calculated by Student’s *t*-test. Categorical variables were calculated by Chi-square analysis except for data marked with §, which were calculated by Fisher’s exact test.

SD standard deviation WBC white blood cell NEUT neutrophil LYM lymphocyte HB hemoglobin RBC red blood cell count PLT platelets ALT alamine aminotransferase AST asparate aminotransferase ALB albumin BUN blood urea nitrogen CREA creatinine UA uric acid Ca calcium ion TT3 total triiodothyronine 3 TT4 total triiodothyronine 4 FT3 free triiodothyronine 3 FT4 free triiodothyronine 4 TSH thyroid stimulating hormone ANTITGAB anti-thyroglobulin antibodies ANTITPOAB anti-thyroid peroxidase antibody TG thyroglobulin URBC urinary red blood cell LNM lymph node metastasis

### US image and US radiomic feature extraction

US examinations of thyroid nodules were performed with high-frequency linear probes (5 MHz to 14 MHz) with a variety of US systems: Philips EPIQ7C (Philips Medical Systems, Best, the Netherlands), GE Volume E8 (GE Medical Systems, Chicago, IL, USA), Siemens ACUSON OXANA 2 (Siemens Medical Solutions, Malvern, PA, USA), Esaote MyLab Class C (Esaote, Genoa, Italy), Hitachi HI VISION Preirus (Hitachi-Aloka Medical, Tokyo, Japan) and Mindray Resona 7T (Mindray Medical International, Shenzen, China). The US images included both transverse and longitudinal section of nodules and were saved as JPG images. Two US experts (YY, HL) who had 24 and 16 years of experience in in performing thyroid US examination retrospectively reviewed the images. They scored the TI-RADS of each nodule and draw the region of interest (ROI) of it without knowing the states of LNM. Before and in the middle of the study, they analyzed 20 nodules jointly to establish a standard. Finally, they reviewed half of the nodules, respectively.

The US images of the largest cross section were obtained and the ROI was manually delineated by Paint Win10 for windows. US radiomic features were extracted by an open-source software (Pyradiomics, http://pyradiomics.readthedocs.io/en/latest/index.html) (Jiang et al., 2020) (Fig. 2). In order to reduce image noise and increase the contrast of structures of interest, histogram equalization algorithm was employed before normalization. The scale within the ROI was normalized from 0 to 255. The binWidth of image was 25. Resample was skipped for the two-dimensional nature of US image. A total of 1,769 candidate US radiomic features were extrated for each nodule. There were nine shape features, 360 first-order statistical features and 1,400 textural features. The gray matrices in textural features included gray-level size-zone matrix (GLSZM), gray-level co-occurrence matrix (GLCM), gray-level dependence matrix (GLDM) and gray-level runlength matrix (GLRLM). In addition, high-dimensional features were acquired by filters including square, square root, Laplacian of Gaussian (LOG) with different sigma values (1.0 mm–10.0 mm with step 1.0 mm), wavelet with 2D transform (low-pass/low-pass, LL; low-pass/high-pass, LH; high-pass/low-pass, HL; high-pass/high-pass, HH), logarithm, gradient and exponential (Fig. 2).

### Predicting model building and validating by ML

All nodules were divided into training group (282 nodules) and validation group (118 nodules) at random by a ratio of 7:3 (Fig. 2). The 1803 features contained 34 clinical characteristics and 1,769 US radiomic features. And the number of non-LNM and LNM was 245 and 155, which were not class balanced. The number of non-LNM and LNM for training group and validation group was 165 and 117, 80 and 38, respectively. Their ratio was 1.4 and 2.1 for training group and validation group. In training group, all features were selected using randomforest (RF) and the least absolute shrinkage and selection operator (LASSO) regression after standardization. To avoid over-fitting and select the most significant features, five-fold and seven-fold cross-validation was used for RF and LASSO regression, respectively (S1, S3). Parameter tuning was employed to improve the performance of them (to have the minimal error rate for RF and the minimal lambda for LASSO regression). The top-100 features were selected according to the value of mean decrease of accuracy (MDA) and the result of cross-validation by RF (Data S1, S2). And 23 features were selected by LASSO regression (Data S2, S3, S4). Then five classifiers were separately used for data1 and data2. The classifiers were RF, k nearest neighbors (KNN), binary logistics regression (LR), support vector machine (SVM) and the extreme gradient boosting (xgboost). Parameter tuning was conducted for the best performance of each model. In total, 10 models were developed and their predictive performance were compared by the area under the receiver operating characteristic (ROC) curve (AUC), sensitivity, specificity, accuracy, positive predictive value (PPV), negative predictive value (NPV), balanced accuracy and kappa value. Cross-validation was employed for best model. The workflow of model building and validating was shown in Fig. 2.

### Statistical analysis

The normality analysis, Student’s *t*-test, Mann–Whitney U test, Chi-square analysis and Fisher’s exact test were performed with SPSS software (version 19.0, IBM). The random allocation, RF, LASSO regression, binary LR, KNN, SVM, xgboost, delong’s test, Confusion Matrix and other statistical analysis were carried out with R software (version 4.0.3; R Core Team, 2020). The package for RF, KNN, SVM, xgboost LASSO regression and binary LR was “randomForest”, “kknn”, “e1071”, “xgboost”, “glmnet” and “glmnet”. The type for the former two was “classification”. The type and kernel for SVM was “C-classification” and “polynomial”. The booster and object for xgboost was “gbtree” and “rank:pairwise”. The family setting of LASSO regression and binary LR was “binomial()”. A value of *p* < 0.05 was considered statistically significant.

## Results

### Clinical characteristics

The clinical characteristics of nodules in training and validation group were summarized in Table 1. The number of nodules with cervical LNM accounted for 41.49% (117/282) and 32.20% (38/118) of training and validation group, respectively. No significant differences were seen between the two groups except for HB and echogenicfoci (*p* = 0.032, 0.025). The images acquired by Esaote MyLab Class C, Hitachi HI VISION Preirus, Philip EPIQ7C, Siemens ACUSON OXANA 2, GE Volume E8 and Mindray Resona 7T accounted for 59.25% (237/400), 7.75% (31/400), 15.75% (63/400), 12.50% (50/400), 1.25% (5/400) and 3.50% (14/400).

### Feature selection and model performance evaluation

**Figure 3:**
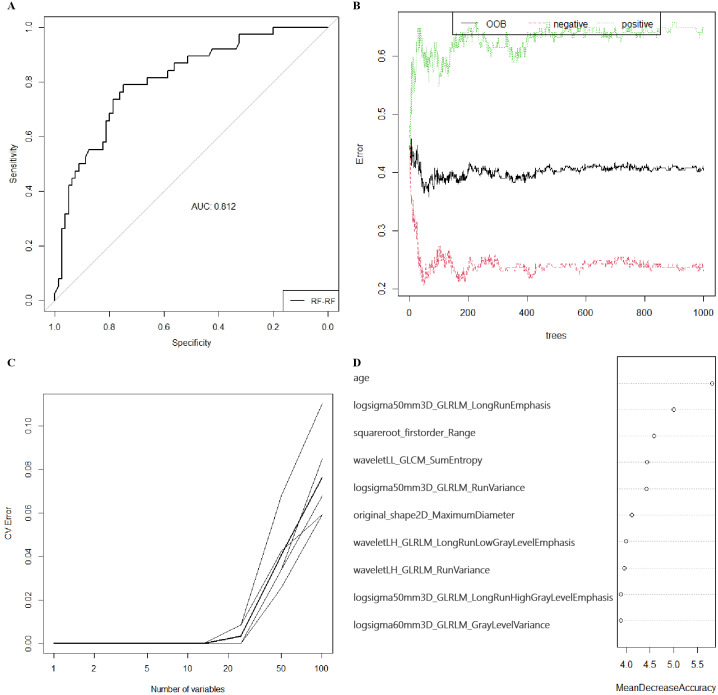
The preoperative predicting value of models and the selection of features of importance. (A) The ROC curve and AUC value for RF-RF model in validation group. (B) Error convergence curve according to the number of trees used in the RF-RF model in training group. (C) Error convergence curve according to the number of variables in the RF-RF model based on 5-fold cross-validation in validation group. (D) Importance score of top 10 variables of RF-RF model with MDA. ROC, the receiver operating characteristic; AUC, area under the curve; RF, randomforest; MDA, mean decrease of accuracy; xgboost, the extreme gradient boosting; KNN, k nearest neighbors; LR, logistics regression; SVM, support vector machine; LASSO, the least absolute shrinkage and selection operator; OOB, out-of-bag; CV, cross-validation; logsigma, Laplacian of Gaussian with sigma; GLRLM, gray-level runlength matrix; GLCM, gray-level co-occurrence matrix; LL, low-pass/low-pass; LH, low-pass/high-pass.

**Figure 4:**
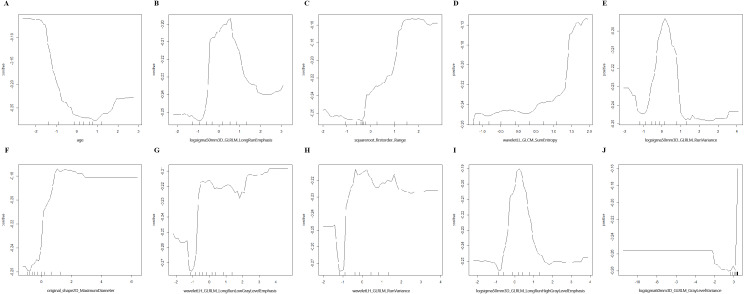
Partial dependence plot on the top 10 variables of RF-RF model. (A–J) Partial dependence plot on age, logsigma5.0mm3D_GLRLM_LongRunEmphasis, squareroot_firstorder_Range, waveletLL_GLCM_SumEntropy, logsigma5.0mm3D_GLRLM_RunVariance, original_shape2D_MaximumDiameter, waveletLH_GLRLM_LongRunLowGrayLevelEmphasis, waveletLH_GLRLM_RunVariance, logsigma5.0mm3D_GLRLM_LongRunHighGrayLevelEmphasis, logsigma6.0mm3D_GLRLM_GrayLevelVariance. RF, randomforest; logsigma, Laplacian of Gaussian with sigma; GLRLM, gray-level runlength matrix; GLCM, gray-level co-occurrence matrix; LL, low-pass/low-pass; LH, low-pass/high-pass.

After standardization, 100 features out of 1803 were selected using RF (MDA >1.744) and 23 features out of 1803 were selected by LASSO regression in training group (S1-S4). Then, RF, KNN, binary LR, SVM and xgboost were used for model construction based on them (Fig. 2). In validation group, the AUC of the 10 models ranged from 0.580−0.812, in which RF-RF model reached the highest and RF-SVM model the lowest (Fig. 3A, Table 2). Delong’s test was carried out for AUC comparison between RF-RF model and other models. The *p* value revealed a significantly higher AUC of RF-RF model than others except for RF-xgboost model (Table 2). The accuracy, sensitivity, specificity, PPV and NPV of these models were shown in Table 2. Among them, RF-RF model had the highest accuracy compared to other models. Since our data was class imbalanced, we calculated the balanced accuracy using Confusion Matrix of each model. The balanced accuracy of RF-xgboost was higher than that of RF-RF model. And the balanced accuracy of RF-RF was similar to the imbalanced one (Table 2). Besides, the kappa value of models in validation group revealed a moderate consistency for RF-RF model and RF-xgboost model (Table 2). We then chose RF-RF as the best model for its highest AUC and relatively high balanced accuracy. Figure 3B showed the modeling process of RF-RF model. And we used 5-fold cross-validation to prove the reliability of RF-RF model and help selecting the most important features (Fig. 3C, S5). Finally, the top 10 variables were chosen with their MDA and partial dependent plot were shown in Figs. 3D and 4A–4J. The selected features included age, 6 GLRLM, 1 GLCM, 1 shape feature, 1 first-order feature, in which 8 were high-dimensional features.

**Table 2:** Comparison of diagnostic performance for different models in validation group.

	Accuracy (95% CI)	Sensitivity	Specificity	PPV	NPV	AUC	*P* value	kappa	Balanced Accuracy
RF-RF	0.7542(0.6665,0.8288)	0.6842	0.7875	0.6047	0.8400	0.812	–	0.4560	0.7359
RF-xgboost	0.7373(0.6483,0.8140)	0.7125	0.7895	0.8769	0.5660	0.787	0.6722	0.4548	0.7510
RF-KNN	0.7034(0.6123,0.7839)	0.6579	0.7250	0.5319	0.8169	0.691	0.0092	0.3605	0.6914
RF-binary LR	0.5508(0.4566,0.6425)	0.5789	0.5375	0.3729	0.7288	0.592	0.0025	0.1017	0.5582
RF-SVM	0.5763(0.4819,0.6667)	0.6053	0.5625	0.3966	0.7500	0.580	0.0009	0.1474	0.5839
LASSO-RF	0.6610 (0.5681,0.7456)	0.5526	0.7125	0.4773	0.7703	0.702	0.0311	0.2546	0.6326
LASSO-SVM	0.6441 (0.5507,0.7300)	0.7632	0.5875	0.4677	0.8393	0.662	0.0244	0.3008	0.6753
LASSO-xgboost	0.6186 (0.5247,0.7065)	0.6625	0.5263	0.7465	0.4255	0.647	0.0147	0.1778	0.5944
LASSO-KNN	0.6949 (0.6034,0.7763)	0.4474	0.8125	0.5312	0.7558	0.630	0.0016	0.2711	0.6299
LASSO-binary LR	0.5593 (0.4650,0.6506)	0.6842	0.5000	0.3939	0.7692	0.627	0.0086	0.1544	0.5921

**Notes.**

CI confidence interval PPV positive predictive value NPV negative predictive value AUC area under curve RF randomforest xgboost the extreme gradient boosting KNN k nearest neighbors LR logistics regression SVM support vector machine LASSO the least absolute shrinkage and selection operator

## Discussion

High-dimensional omics data sets, whose number of variables is much larger than the number of individuals, are usually sparse regarding relevant information. The utilizing of them is often based on ML, a promising computation approaches for classification and regression. RF, one of the ensemble machine learning methods based on decision trees, is a well-suited method for tackling the problem (Degenhardt, Seifert & Szymczak, 2019). It’s widely applied to analyzing data from life science. And the availability of variable importance measures (VIMs) accounts for its widespread (Nembrini, Konig & Wright, 2018; Park et al., 2020a). Impurity importance (often called Gini importance) and the permutation importance (also known as MDA) are the most widely used VIMs. Literatures showed that the Gini importance had bias while permutation importance didn’t (Degenhardt, Seifert & Szymczak, 2019; Nembrini, Konig & Wright, 2018). Thus, we chose features according to their MDA during feature selection by RF. LASSO regression is commonly used to reduce variables recruited into model. Its algorithm could process multicollinearity data, predict and select variables with biased estimate. Besides, it could overcome the multicollinearity problem when doing regression analysis (Chen et al., 2020). With the widely used classifier of RF, KNN, SVM, binary LR and xgboost, the ML process could help dealing our high-dimensional data sets and find the most important features for cervical LNM predicting of PTC.

Literature showed that cervical LNM negatively impacted patients’ overall survival and disease-free survival in PTC patients. It associated with higher rate of distant metastasis and disease-related mortality by 11.2-fold and 3-fold, respectively (Guo et al., 2019; Hartl et al., 2012; Zhou et al., 2020a). However, the detection rate of cervical LNM using US was unsatisfying, especially for central LNM. Besides, prophylactic cervical lymph node dissection could potentially lead to nerve injury and hypoparathyroidism (Guo et al., 2019; Liu et al., 2018). Therefore, preoperative prediction of cervical LNM was essential for all patients. Generally, US features like tumor size, echogenicity, “wider-than-taller” shape, extrathyroidal extension and calcification, clinical information like age were proved indicators of cervical LNM. However, because the expertise of the operator significantly influenced the diagnostic accuracy, these results only served as reference in clinical practice (Guo et al., 2019; Liu et al., 2018; Liu et al., 2019).

With the development of radiomics analysis, several articles revealed a promising result for preoperative prediction of cervical LNM for PTC nodules. Liu introduced US radiomic analysis to preoperative cervical LNM prediction and proved that LNM was associated with larger size, younger age, irregular tumor shape, obscure boundary, spiculate margin, taller-than-wide shape, calcification, complex echo pattern, thyroid invasion and posterior region homogeneity (AUC in validation group = 0.782) (Liu et al., 2019). In addition, Cui and Jiang revealed an AUC of 0.90 and 0.83 for cervical LNM prediction in the radiomics signature based on strain elastography ultrasound images and shear-wave elastography images. However, only Jiang’s study detected the predictive value of high-dimensional features and showed the wavelet transform of B-mode images were related with cervical LNM (Jiang et al., 2020; Liu et al., 2018).

In the present study, with the help of ML, we established 10 models based on clinical characteristics and US radiomic features for preoperative prediction of cervical LNM in PTC nodules. The AUC, accuracy and balanced accuracy for each model varied in validation group and RF-RF model was proved the best one among them. After cross-validation, RF-RF model selected the top 10 features of importance, which included age, six GLRLM, 1 GLCM, 1 shape feature and 1 first-order feature. Among them, four features were LOG based features, three were wavelet based feature and 1 were square root based feature. Unlike our study, Zhou et al. (2020a) demonstrated an ultrasound radiomics nomogram for central LNM with an AUC of 0.85 and their equation included age, TPOAB level, TG level, radiomic signature and ultrasonography-reported lymph node status. Because the radiomic features were extracted based on parameters mentioned by different guidelines, they lacked higher-dimensional features. Besides, Tong et al. (2020) established a nomogram for lateral LNM with an AUC of 0.91. They included six textural features (five GLSZM and one GLCM) in the equation with little higher-dimensional features, either.

Shape features described the shape of tumor volume, along with its geometric properties. For voxels within tumor volume, first-order features depicted their distribution of intensities while textural features measured their inter-relationship of distributions (Nazari et al., 2020). The GLRLM quantified the length of consecutive pixels of the same gray level value in images, while GLCM represented the number of times specific combination of gray levels occurred in two separated pixels (Nawabi et al., 2020). LOG filter acted as a combination of Laplacian operator and Gaussian filter, which might detect edges as well as noise in a smoothed image for filtering and differentiation (Dinapoli et al., 2018; Shayesteh et al., 2020; Suo et al., 2016). It was employed for image filtering in the spatial domain and was widely used in radiomics in literature (Dinapoli et al., 2018; Suo et al., 2016). Different filter sigma parameters applied for fine or coarse anatomic details for textural features (Shayesteh et al., 2020; Suo et al., 2016). According to literature, wavelet filter could enhance certain characteristics based on its frequency domain in images and was widely used in image compression and preprocessing (Chaddad, Daniel & Niazi, 2018; Vuong et al., 2019). Besides, square root filter could improve the overall condition of covariance matrices by improving their update accuracy and avoiding the negative definiteness (Caruso et al., 2017; Wei et al., 2018). The LOG-, wavelet- and square root-filtered features selected by our result deeply implied the importance of including higher-dimensional statistical methods, meanwhile highlighting their unique role for radiomics analysis.

There were several limitations in our study. Firstly, due to the retrospective nature, the clinical procedure was not strict and some nodules had incomplete clinical data. In addition, the number of samples was relatively small. These conditions could reduce the number of included nodules and may lead to the class imbalance of our data. Furthermore. We did not re-balance data at the beginning of statistical analysis. Although the ratio of non-LNM to LNM was mildly and we used multi-method for validation, such as the calculation of AUC and balanced accuracy from Confusion Matrix. The balanced accuracy was slightly lower than imbalanced accuracy. Thus, larger cohorts, more strict clinical procedure and re-balancing techniques were required in the future. Secondly, some of the recorded US images were discrete ones which might cause the capture of unrepresentative portion of the tumor, leading to the inconsistency of data. Thus, more strict clinical procedure or prospective study with rules could be carried out in the future. Thirdly, models were validated in mono-center cohort without additional test group. However, the independent validation group, which did not involve in feature selection and model construction, could offer reliable results. Multi-methods comparison and cross-validation further proved its reliability. An additional test group from another center was still needed for more convincing results. Thus, multi-center test group for validation should be carried out in the future.

## Conclusions

In conclusion, our study, for the first time, established preoperative predicting models with the help of ML for cervical LNM based on clinical characteristics and US radiomic features. They were expected to help with diagnosis, recurrence prediction and treatment decision of PTC.

##  Supplemental Information

10.7717/peerj.14546/supp-1 Data S1 Raw data All clinical information and ultrasonic radiomics data for nodules.

10.7717/peerj.14546/supp-2 Supplemental Information 2 Supplemental Figures and Tables

10.7717/peerj.14546/supp-3 Supplemental Information 3 Codebook for raw data WBC, white blood cell; NEUT, neutrophil; LYM, lymphocyte; HB, hemoglobin; RBC, red blood cell count; PLT, platelets; ALT, alamine aminotransferase; AST, asparate aminotransferase; ALB, albumin; BUN, blood urea nitrogen; CREA, creatinine; UA, uric acid; Ca, calcium ion; TT3, total triiodothyronine 3; TT4, total triiodothyronine 4; FT3, free triiodothyronine 3; FT4, free triiodothyronine 4; TSH, thyroid stimulating hormone; ANTITGAB, anti-thyroglobulin antibodies; ANTITPOAB, anti-thyroid peroxidase antibody; TG, thyroglobulin; URBC, urinary red blood cell; LNM, lymph node metastasis.

## Additional Information and Declarations

### Data Availability

The following information was supplied regarding data availability: The raw measurements are available in the Supplementary File.
