# Supplementary material for: Models of ultrasonic radiomics and clinical characters for lymph node metastasis assessment in thyroid cancer: a retrospective study
Source: PeerJ. 2023 Jan 12;11:e14546. doi: 10.7717/peerj.14546 (PMC9840861; doi:10.7717/peerj.14546)
Supplement: Supplemental Information 2 [file peerj-11-14546-s002.docx]

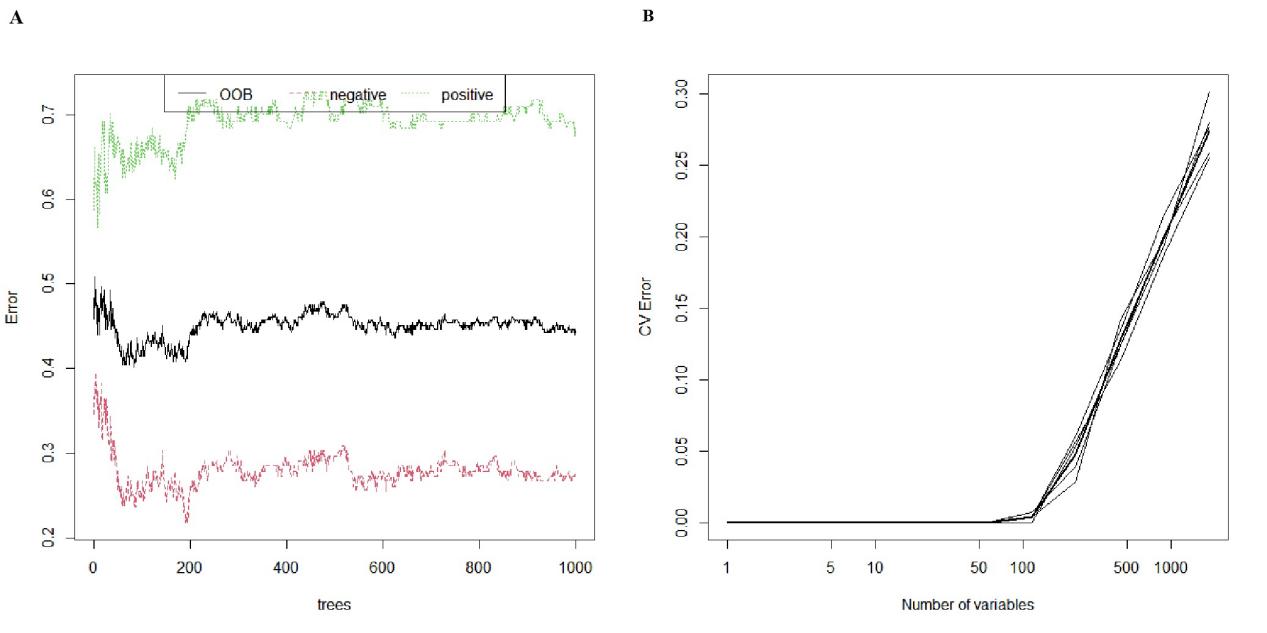


**S1.** Selection of LNM-associated features using RF in building models. (A) Error convergence curve according to the number of trees used in the RF model in training group. (B) Error convergence curve according to the number of variables in the RF model based on 5-fold cross-validation in training group. LNM, lymph node metastasis; RF, randomforest; OOB, out-of-bag; CV, cross-validation.

**S2.** The selected features of RF model based on Mean Decrease Accuracy value.

| Features | Mean Decrease Accuracy |
| --- | --- |
| logsigma70mm3D_GLCM_DifferenceVariance | 3.442338719 |
| logsigma100mm3D_GLCM_Idn | 3.294362441 |
| logsigma50mm3D_GLRLM_LongRunEmphasis | 2.907933971 |
| logsigma100mm3D_GLCM_Imc1 | 2.848503234 |
| logsigma50mm3D_GLCM_JointEntropy | 2.700642897 |
| logsigma50mm3D_GLCM_Idn | 2.680285674 |
| logsigma40mm3D_GLDM_DependenceNonUniformityNormalized | 2.649375099 |
| squareroot_GLSZM_SmallAreaLowGrayLevelEmphasis | 2.561607438 |
| logsigma30mm3D_GLSZM_SizeZoneNonUniformity | 2.515200473 |
| age | 2.469885086 |
| waveletLL_GLSZM_SmallAreaHighGrayLevelEmphasis | 2.415871739 |
| waveletLL_GLCM_SumEntropy | 2.403451695 |
| logsigma30mm3D_GLSZM_LargeAreaHighGrayLevelEmphasis | 2.388607208 |
| logsigma60mm3D_GLRLM_GrayLevelVariance | 2.385591031 |
| logsigma50mm3D_GLRLM_RunVariance | 2.385295934 |
| logsigma50mm3D_GLCM_SumEntropy | 2.381945955 |
| logsigma90mm3D_GLCM_Imc2 | 2.37975256 |
| waveletLH_GLSZM_SizeZoneNonUniformity | 2.351606132 |
| logarithm_firstorder_Median | 2.344617418 |
| logsigma60mm3D_GLCM_Idm | 2.340143257 |
| exponential_firstorder_Variance | 2.334027244 |
| waveletHH_GLRLM_LongRunLowGrayLevelEmphasis | 2.313033086 |
| square_GLRLM_ShortRunLowGrayLevelEmphasis | 2.310312281 |
| logsigma20mm3D_GLRLM_LongRunHighGrayLevelEmphasis | 2.300114726 |
| logsigma50mm3D_GLRLM_LongRunHighGrayLevelEmphasis | 2.258299248 |
| ANTITGAB | 2.257590595 |
| logsigma40mm3D_GLRLM_LongRunHighGrayLevelEmphasis | 2.236490899 |
| logsigma40mm3D_GLDM_DependenceVariance | 2.222274931 |
| squareroot_firstorder_Range | 2.210056901 |
| waveletHL_firstorder_Entropy | 2.20493006 |
| logsigma30mm3D_GLSZM_SmallAreaEmphasis | 2.166425562 |
| logsigma100mm3D_GLDM_SmallDependenceEmphasis | 2.161927299 |
| logsigma50mm3D_GLRLM_HighGrayLevelRunEmphasis | 2.149818249 |
| logsigma40mm3D_GLSZM_ZonePercentage | 2.146638329 |
| square_GLDM_DependenceNonUniformity | 2.115328511 |
| waveletLH_GLDM_DependenceNonUniformity | 2.113110565 |
| logsigma70mm3D_GLCM_Correlation | 2.085284956 |
| logsigma20mm3D_firstorder_Mean | 2.062195706 |
| logarithm_GLCM_Imc1 | 2.058154579 |
| logsigma70mm3D_GLSZM_LargeAreaEmphasis | 2.05195445 |
| waveletLL_GLSZM_GrayLevelVariance | 2.050627697 |
| logsigma40mm3D_GLCM_Imc1 | 2.027466223 |
| logsigma60mm3D_GLCM_MCC | 2.026707168 |
| waveletLL_GLCM_Correlation | 2.021495861 |
| originalshape2D_Maximum_Diameter | 2.015011187 |
| logsigma90mm3D_firstorder_InterquartileRange | 2.012767863 |
| original_shape2D_Elongation | 2.006399302 |
| squareroot_GLCM_SumEntropy | 1.99906682 |
| logsigma60mm3D_firstorder_Median | 1.985804489 |
| logsigma60mm3D_firstorder_10Percentile | 1.984271372 |
| logsigma80mm3D_GLCM_SumEntropy | 1.982301874 |
| logsigma20mm3D_GLRLM_RunEntropy | 1.980886173 |
| logarithm_firstorder_Mean | 1.964682756 |
| logsigma90mm3D_GLSZM_GrayLevelNonUniformity | 1.960297904 |
| logsigma40mm3D_firstorder_TotalEnergy | 1.957262258 |
| original_firstorder_TotalEnergy | 1.952041398 |
| original_GLRLM_RunLengthNonUniformityNormalized | 1.950707237 |
| squareroot_GLSZM_HighGrayLevelZoneEmphasis | 1.946934304 |
| waveletLH_GLCM_JointEnergy | 1.938152843 |
| waveletLH_GLRLM_RunVariance | 1.932361515 |
| waveletHH_GLRLM_LongRunEmphasis | 1.931604791 |
| waveletLL_GLCM_Imc2 | 1.923805337 |
| logsigma80mm3D_firstorder_RobustMeanAbsoluteDeviation | 1.919619001 |
| logsigma20mm3D_firstorder_10Percentile | 1.915172116 |
| logsigma60mm3D_GLRLM_RunPercentage | 1.911094116 |
| waveletHL_firstorder_Median | 1.899101544 |
| logsigma10mm3D_GLSZM_LargeAreaEmphasis | 1.898739837 |
| logsigma30mm3D_GLDM_DependenceNonUniformityNormalized | 1.894202084 |
| logsigma20mm3D_firstorder_TotalEnergy | 1.884635295 |
| squareroot_GLRLM_ShortRunLowGrayLevelEmphasis | 1.882161421 |
| logsigma70mm3D_GLCM_JointEntropy | 1.879656003 |
| exponential_GLSZM_LargeAreaEmphasis | 1.878771427 |
| logsigma60mm3D_GLRLM_LongRunLowGrayLevelEmphasis | 1.875265523 |
| logsigma10mm3D_GLCM_ClusterTendency | 1.87116524 |
| logsigma80mm3D_firstorder_Maximum | 1.865183301 |
| logsigma100mm3D_GLCM_Idmn | 1.864642734 |
| logsigma80mm3D_GLRLM_RunLengthNonUniformityNormalized | 1.852966822 |
| logsigma40mm3D_GLRLM_LongRunEmphasis | 1.835899894 |
| logarithm_GLSZM_GrayLevelNonUniformityNormalized | 1.834274717 |
| logsigma80mm3D_GLCM_JointEntropy | 1.833734197 |
| logsigma30mm3D_GLCM_InverseVariance | 1.829201456 |
| waveletLH_GLRLM_LongRunLowGrayLevelEmphasis | 1.825475796 |
| waveletLH_firstorder_Maximum | 1.82280215 |
| logsigma60mm3D_firstorder_90Percentile | 1.821766788 |
| logsigma50mm3D_GLDM_LargeDependenceEmphasis | 1.817627623 |
| squareroot_GLCM_SumAverage | 1.814586705 |
| exponential_GLDM_DependenceNonUniformity | 1.814045565 |
| original_GLSZM_LargeAreaHighGrayLevelEmphasis | 1.810612012 |
| waveletHL_firstorder_RobustMeanAbsoluteDeviation | 1.802476149 |
| logsigma30mm3D_GLCM_SumSquares | 1.800811751 |
| logsigma10mm3D_GLSZM_GrayLevelVariance | 1.798159556 |
| waveletLL_GLSZM_HighGrayLevelZoneEmphasis | 1.785759743 |
| gradient_firstorder_Energy | 1.782318805 |
| logsigma50mm3D_GLCM_DifferenceVariance | 1.770452141 |
| original_GLSZM_SmallAreaEmphasis | 1.761187711 |
| logarithm_firstorder_InterquartileRange | 1.760621506 |
| logsigma90mm3D_GLRLM_ShortRunHighGrayLevelEmphasis | 1.760161341 |
| waveletHL_GLSZM_SmallAreaHighGrayLevelEmphasis | 1.753846028 |
| original_GLRLM_GrayLevelNonUniformityNormalized | 1.749520871 |
| logsigma80mm3D_GLCM_InverseVariance | 1.744447008 |

Footnote: RF, randomforest; GLSZM, gray-level size-zone matrix; GLCM, gray-level co-occurrence matrix; GLDM, gray-level dependence matrix; GLRLM, gray-level runlength matrix; LL, low-pass/low-pass; LH, low-pass/high-pass; HL, high-pass/low-pass; HH, high-pass/high-pass; ANTITGAB, anti-thyroglobulin antibodies.


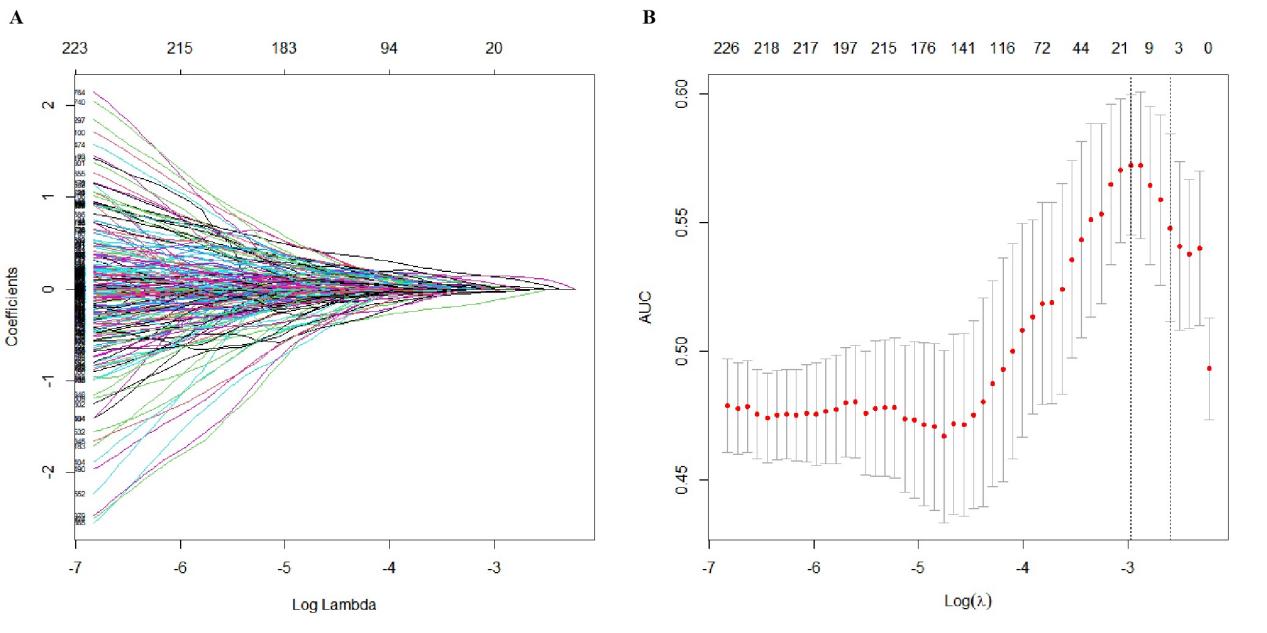


**S3.** Selection of LNM-associated features using LASSO regression in building models. (A) The coefficient profiles of 1804 features against the log Lambda (λ) when building models. (B) Tuning parameter (λ) selection in the LASSO logistic model based on 7-fold cross-validation. LNM, lymph node metastasis; LASSO, the least absolute shrinkage and selection operator; AUC, area under the curve.

**S4.** The coefficients of selected features of LASSO logistic model.

| Features | Coefficients |
| --- | --- |
| size | -0.3511604 |
| sex | 0.02778425 |
| composition | -0.1209118 |
| echogenicfoci | 0.1278081 |
| TT3 | 0.05224362 |
| Logarithm_firstorder_Kurtosis | 0.000599087 |
| Logarithm_firstorder_RobustMeanAbsoluteDeviation | 0.1411578 |
| Square_GLRLM_RunLengthNonUniformityNormalized | 0.01784644 |
| Exponential_GLRLM_RunLengthNonUniformityNormalized | 1.92759E-16 |
| Gradient_GLRLM_RunLengthNonUniformityNormalized | 2.40948E-17 |
| WaveletLL_GLSZM_GrayLevelVariance | -0.03316834 |
| WaveletLL_GLSZM_HighGrayLevelZoneEmphasis | 2.13783E-14 |
| WaveletLL_GLSZM_LargeAreaEmphasis | 0.02457482 |
| WaveletLL_GLSZM_SizeZoneNonUniformity | -1.20231E-14 |
| Square_GLSZM_SmallAreaEmphasis | 0.00189928 |
| Square_GLSZM_SmallAreaHighGrayLevelEmphasis | -0.01868737 |
| Square_GLSZM_SmallAreaLowGrayLevelEmphasis | -1.1082E-15 |
| Square_GLSZM_ZoneEntropy | -1.64821E-16 |
| Exponential_GLSZM_SmallAreaHighGrayLevelEmphasis | -5.03261E-18 |
| Exponential_GLSZM_SmallAreaLowGrayLevelEmphasis | -1.50978E-17 |
| Gradient_GLSZM_SmallAreaHighGrayLevelEmphasis | -5.03261E-18 |
| logsigma20mm3D_GLSZM_SmallAreaEmphasis | -0.02484 |
| logsigma90mm3D_GLSZM_SizeZoneNonUniformityNormalized | 0.000232344 |

Footnote: LASSO, the least absolute shrinkage and selection operator; GLSZM, gray-level size-zone matrix; GLRLM, gray-level runlength matrix; LL, low-pass/low-pass; TT3, total triiodothyronine 3.


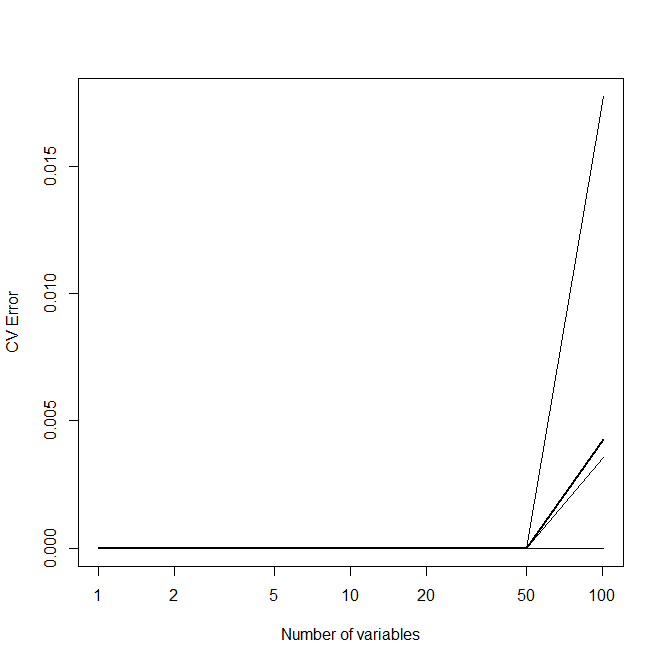


**S5.** Error convergence curve according to the number of variables in the RF-RF model based on 5-fold cross-validation in training group. RF, randomforest; CV, cross-validation.
